# Deep learning-based skin lesion analysis using hybrid ResUNet++ and modified AlexNet-Random Forest for enhanced segmentation and classification

**DOI:** 10.1371/journal.pone.0315120

**Published:** 2025-01-16

**Authors:** Saleem Mustafa, Arfan Jaffar, Muhammad Rashid, Sheeraz Akram, Sohail Masood Bhatti

**Affiliations:** 1 Faculty of Computer Science & Information Technology, The Superior University, Lahore, Pakistan; 2 Intelligent Data Visual Computing Research (IDVCR), Lahore, Pakistan; 3 Department of Computer Science, National University of Technology, Islamabad, Pakistan; 4 Information Systems Department, College of Computer and Information Sciences, Imam Mohammad Ibn Saud Islamic University (IMSIU), Riyadh, Saudi Arabia; University of Sargodha, PAKISTAN

## Abstract

Skin cancer is considered globally as the most fatal disease. Most likely all the patients who received wrong diagnosis and low-quality treatment die early. Though if it is detected in the early stages the patient has fairly good chance and the aforementioned diseases can be cured. Consequently, diagnostic identification and management of the patient at this level becomes a rather enormous task. This paper offers a cutting-edge hybrid deep learning approach of better segmentation and classification of skin lesions. The proposed method incorporates three key stages: preprocessing, segmentation of lesions, and classification of lesions. By the stage of preprocessing, a morphology-based technique takes out hair so as to enhance the segmentation precision to use the cleansed images for subsequent analysis. Segmentation cuts off the lesion from the surrounding skin, giving the classification phase a dedicated area of interest and the ability to clear the background noise that may affect classification rates. The isolation enables the model to better analyze anatomical lesion features in order to achieve accurate benign and malignant classifications. Using ResUNet++, the cutting-edge deep learning architecture, we achieved accurate lesion segmentation. Next, we will modify and use an AlexNet-Random Forest (AlexNet-RF) based classifier for robust lesion classification. The proposed hybrid deep learning model is intensively validated on the Ham10000 data set which is one of the most popular datasets for skin lesions analysis. The obtained results show that the utilized approach, compared to the previous ones, is more effective, giving better segmentation and classification results. This method takes advantage of ResUNet++ strong classification skill and modified AlexNet-Random Forest robustness for more accurate segmentation. There is a high probability that ResUNet++, which is highly proficient at medical image segmentation, can produce better segmentation of lesions than the simpler models. The composition of AlexNet’s extraction of features with Random Forest ability to reduce overfitting possibly may be more precise in the classification when compared to using only one model.

## Introduction

Human skin, repeatedly undefended to environmental toxins, bears to suffer the main part or force of it, making it the utmost common type of cancer. In the UK alone, this translates to roughly 46,000 new cases each year, highlighting the crucial need for awareness and prevention measures. Lesion cancer arises in various forms, with squamous cell carcinoma (SCC) and basal cell carcinoma (BCC) existence the utmost common. Both are classified as non-melanoma and generally have good prognoses. However, melanoma is the most assertive type, demanding untimely detection and treatment. While ultraviolet radiation (UV) from the sun is the source of a problem, individual risk factors like skin tone can play a role. Studies propose that melanin, a natural pigment in the skin, offers some protection averse to sun damage, possibly but not yet actually explaining the higher risk perceive in people with lighter skin tones [1]. Melanoma, a rare but belligerent tumor have its origin in melanin-producing cells, can appear on skin, eyes, and equivalent to internal tissues. in defiance of its low prevalence, its incidence rate outpaces all other skin cancers, emphasize the need for awareness and early detection [2].

Research shows that catching early-stage melanoma can noticeable way to improve outcomes, with one study reporting a 90% reduction in ephemerality. Melanoma’s severity varies outstandingly on all parts of populations, making earmark prevention crucial [3]. Early detection can extremely improve result, with Stage I patients experiencing a 94–98% ten-year survival rate compared to Stage IV’s mere 10–15%. By recognize high-risk groups, we can prioritize awareness and screening attempt, potentially saving lives through timely involvement [4]. The traditional biopsy, extremely important for skin cancer diagnosis, comes with downside. Its interfering nature, pain, and taking a lot of time to process create a impediment to early detection. Enter computer-aided technology, handout a promising solution. By examine skin lesions with modern algorithms, this approach promises a brisk, less painful, and cheaper way to identify skin cancer [5]. Recognizing the censorious gap in early skin cancer detection, this research embarks on a mission to grow a computer-assisted diagnostic system. Our focus lies on Categorizing the dermoscopic images into melanoma class and non-melanoma class, paving the way for a future where accurate and Approachable diagnosis empowers patients and improves results. Motivated by the limitations of current skin lesion analysis techniques, this research seeks to develop a novel and Exact image segmentation method Concretely for skin lesions. This innovative approach aims to Optimize detection and classification Trustworthiness, Facilitating health care professionals with a faster, easier, and more Truthful tool.

Deep learning has potential immense for medical images diagnosis, detection and classification. Recently research shows that Deep Learning has revolution for by proficiently early detection, extracting features from the skin lesion, allowing more accurate segmentation of lesion part from the image and classification Deep learning has ability to learn pattern of characterize and outperform powerfully for skin cancer diagnosis. Deep learning algorithms specially designed for skin cancer. Deep learning algorithm has effective for more efficient and precise for diagnostic tool.

Major contribution in this paper is the inclusion of a morphology-based hair removal technique during preprocessing is a key contribution. Hair can obscure lesion boundaries, and removing it likely improves segmentation accuracy by providing a cleaner image for analysis. This is especially important for accurate feature extraction in the classification stage. The paper leverages ResUNet++, a state-of-the-art deep learning architecture known for its effectiveness in medical image segmentation. By utilizing this powerful tool, the method likely achieves superior lesion segmentation compared to simpler approaches. This translates to a more precise analysis in the classification stage. The proposed methodology utilizes a hybrid deep learning approach, combining AlexNet for feature extraction with a Random Forest classifier. This approach leverages the strengths of both techniques. AlexNet can capture intricate features from the segmented lesions, while the Random Forest classifier improves robustness and reduces overfitting. This combination can potentially lead to more accurate classification compared to using a single model.

## Literature review

This paper [6] introduced a novel approach for Categorizing two form of skin cancer (melanoma and non-melanoma) by Integrating both color and grayscale information, achieving superior results compared to using either alone. The method utilizes k means clustering for Subsetting and Embraces the ABCD criteria (asymmetry, border irregularity, color, diameter) used for feature extraction. The experiment, supervised on 150 images (75 each of melanoma and non-melanoma), Illustrates that Support Vector Classifier (SVC) and 1-Nearest Neighbor (1-NN) achieve the highest Credibility with the chosen feature set. This paper [7] presented a detailed 3D reconstruction algorithm based on 2D images. The process involves pre-processing and binarizing the images, followed by segmentation using the Resilient snake algorithm. To enhance accuracy, the approach Integrates not only shape and RGB information but also a novel 3D depth estimation parameter, leading to improved classification efficiency and ultimately more accurate 3D reconstructions. This paper [8] inspected the potential of shearlet transforms and Naive Bayes classifiers for melanoma classification. The proposed method Thoroughly analyzes the dataset using three different scales of shearlet decomposition (50, 75, and 100 coefficients). These features are then fed into a Naive Bayes classifier, demonstrating optimal accuracy at the 3rd classification level when utilizing 100 coefficients. This paper [9] presented a comprehensive analysis of extraction features methods for skin lesion detection, culminating in a powerful and efficient system. The proposed system implements hair removal and OTSU segmentation, followed by the extraction of various features like circularity, luminance, corners, and skewness. Each feature’s accuracy is meticulously evaluated, with the combination of shape and texture + color features achieving an impressive 97% accuracy, demonstrating its superiority for accurate skin lesion characterization. Clear and precise analysis of key features in skin lesions is crucial for early melanoma detection, which can ultimately save lives [10]. While Leiter U et al. [11] demonstrated promising results for automated lesion classification with an AI achieving 95% sensitivity and 88% specificity, similar to expert dermatologists (95% and 90%, respectively), further research is necessary to optimize and validate these systems before widespread clinical implementation. Machine learning, a rapidly evolving field within artificial intelligence, grants computers the remarkable ability to learn and improve without explicit instructions [12]. In paper [13] had proposed that final layer replaced by SoftMax for automated classification method. This technique got accuracy 98.61%. In paper [14], had suggested to categorize the image into melanoma and nonmelanoma for reliable diagnoses. Deep Learning model ResNet-101 and inception architecture used for classification and got 84% and 87.4% respectively. In paper [15], had presented the FRCNN model’s performance with the training dataset and achieved 86.2%. In paper [16] proposed dilated convolution for classification of skin cancer classification. Four pretrained transfer learning-based CNN models, such as MobileNet, VGG16, VGG19, and InceptionV3. Performance of Dilated InceptionV3 gave more accurate and robust results and achieved accuracy 89.81%. In paper [17], had recommended a deep learning model U-net architecture, which was very effective. In order to more enhance the segmentation of lesion part, three basic post-processing measures were completed that removed loose regions, disconnected unnecessary regions and filled in gaps. This model showed very good results with accuracy is 98.3%. In paper [18], has suggested that FCNs model for segmentation of skin cancer. Proposed network train images from beginning to end by using ground truth label and pixel image as input. This model outclassed from existing state-of-the arts edge techniques for segmenting skin cancer good results. In paper [19], had proposed CNN architecture for dermoscopic images of colored skin lesion. Model is used preprocessing, extraction of features and classification. CNN model outperformed result with accuracy of 76.83%. In paper [20] had proposed MobilNetV3-UNet architecture for segmenting lesions using the trivial encoder decoder. And showed very good results of 95.15% on dataset. In paper [21] presented a deep learning model wide-Shuffle Net for the classification. By calculating entropy base weighting and moment than find area of lesion. after this classifying its type. This model achieved 86.3% accuracy. In paper [22] propose segmentation and classification for dermal lesions that is accurate. For this objective, First, segmented the lesion part of skin cancer, and then deep learning model DenseNet-201 was deployed for features extraction and classify the melanoma. This model showed good result of81.29% accuracy. In paper [23] had Suggested a LinkNet-B7 model for segmentation of skin lesion and noise removal that associations the more important aspects of the Efficient Net and Link Net models that used are before now. It showed that the LinkNet-B7 model outclassed than Link Net models when it came to segmentation of skin cancer and removing noise and showed 95.5% accuracy. In paper [24] used ResUnet50 for classification and get 87 accuracies on dataset Xiangya-Derm. We summarize the existing different technique in Table 1

**Table 1:** Comparison table or existing different technique for skin cancer.

Reference	Year	Method	Data set	Advantage	Accuracy	Disadvantage
**Hosny et al. [13]**	2018	AlexNet	PH2	AlexNet is improved with SoftMax for improved classification	98.61%	Complications are high.
**Demir et al. [14]**	2019	Inceptionv3 and ResNet-101	ISIC-2017	huge number of layers can be trained simply.	84.09% 87.42%	high computational power are required
**Jinnai et al. [15]**	2020	FRCNN	ISIC-2019	classification accuracy was higher by FRCNN	86.2%	expensive in training and more time.
**M. Aminur et al. [16]**	2020	Different CNN models InceptionV3	ISIC-2019	Segmentation better performed than another model	89.81%	Only segmentation performed and need for classification with better results
**Araujo et al. [17]**	2021	UNet	PH2	Show more accuracy rate	98.3%	inequalities of data not improve and performance of classification.
**Adegun et al. [18]**	2019	FCN	HAM1000	survival rate of patients is increase	90%	Cost of implementation is very high.
**P. Naronglerdrit et al. [19]**	2019	CNN	HAM1000	More and better effective processing model.	76.83%	Lot of data is required for training
**A. Wibowo et al. [20]**	2021	MobileNetV3-UNet	PH2	accuracy rate is better	88%	No accuracy mention, and need for classification
**M. A. Al-masni et al. [22]**	2020	DenseNet-201	ISIC-2017	Better and more effective	81.29%	
**C. Akyel et al. [23]**	2022	Wide-shuffleNet	HAM1000	classification accuracy was higher on large datasets	86.33%	Need to improvement the in model
**Chan et al. [23]**	2022	Link_Net-B7	ISIC-2000	removes human error in diagnosis of cancer	95.72%	Lot of data is required for training
**Inthiyaz et al. [24]**	2023	CNN ResNet-50	Xiangya-Derm	more number of layers can be trained	87%	Can not generalized on large data

## Proposed methodology

**Fig 1:**
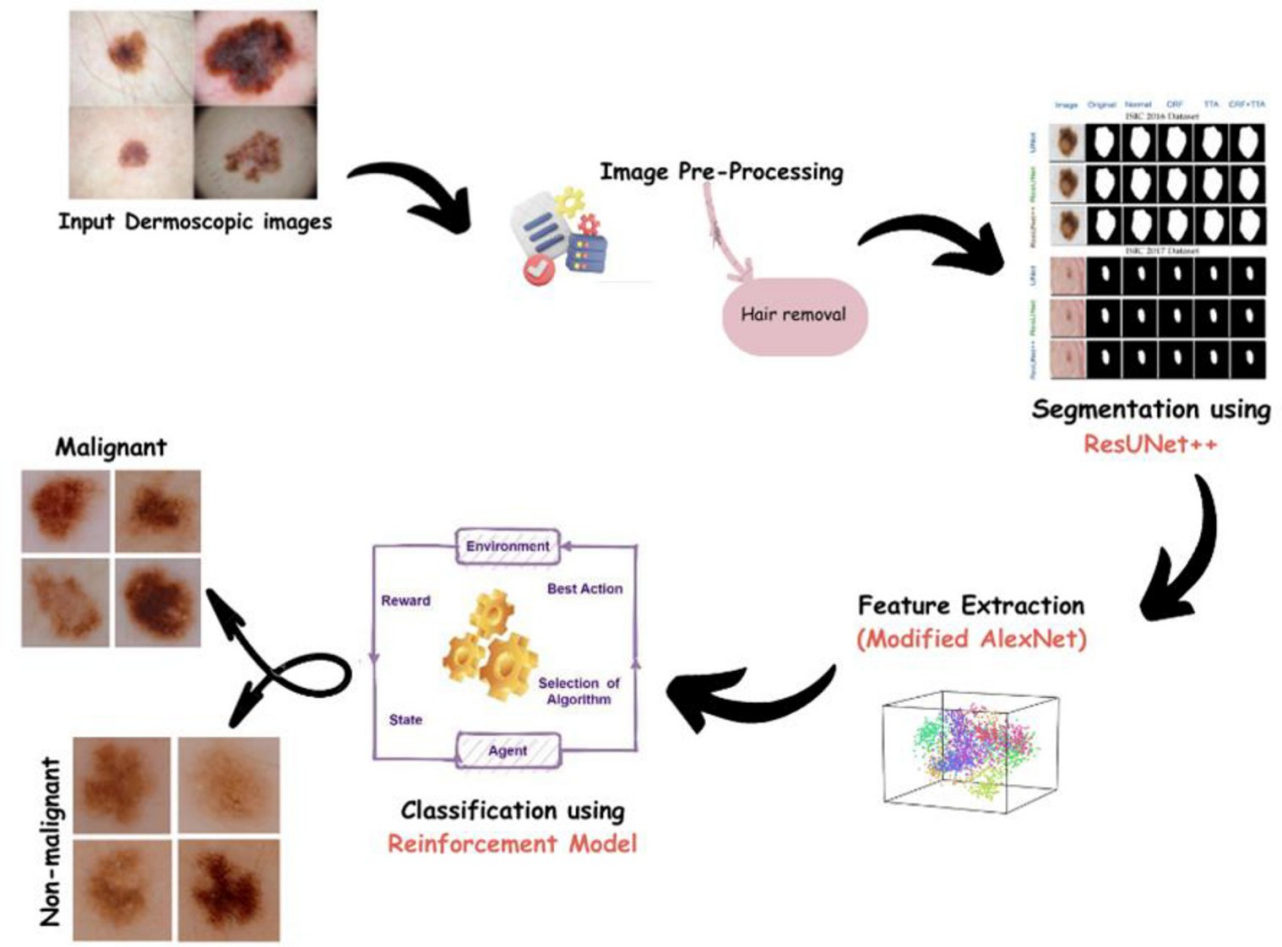
Block diagram of proposed method.

This section explains a novel methodology for skin cancer segmentation and classification using a hybrid deep learning approach has been proposed. The proposed method consists of three key phases: preprocessing, lesion segmentation, and classification as shown in Fig 1. Here we can obstruct lesion boundaries. A morphology-based hair removal technique likely improves segmentation accuracy by creating a cleaner image for analysis compared to methods that don’t address hair. So during preprocessing, a morphology-based technique is employed for efficient hair removal. Segmentation isolates the lesion from surrounding healthy skin. This allows the classification stage to focus solely on the relevant region, eliminating background noise and irrelevant features that could impact classification accuracy. By separating the lesion, the model can analyze its characteristics (color, texture, borders) more precisely for accurate classification as benign or malignant. Accurate segmentation ensures these features are extracted only from the lesion itself, leading to a more reliable feature set for the classification model. ResUNet++, an advanced deep learning framework, is implemented to segment lesions with precision. Stable lesion classification is accomplished subsequent to segmentation through the utilization of an altered iteration of the AlexNet-Random Forest (AlexNet-RF) classifier. A comprehensive assessment of the proposed hybrid deep learning model is performed utilizing the Ham10000 benchmark dataset for skin lesion analysis. The results obtained indicate that the proposed strategy exhibits superior performance in tasks encompassing both segmentation and classification when compared to existing approaches.

It achieves superior lesion segmentation performance compared to simpler approaches by capitalizing on ResUNet++, a widely recognized deep learning framework recognized for its precision in segmenting medical images. Consequently, this leads to a more accurate analysis of categorization of data. The combination of the Random Forest classifier with AlexNet, a stable deep learning model constructed for research on feature extraction, serves to boost the efficiency of both approaches. AlexNet has the capability to capture intricate features, while the implementation of Random Forest improves resilience and reduces overfitting, potentially leading to a more accurate classification than when a single model is employed.

### Morphology-Hough transform based hair removal from skin images

**Fig 2:**
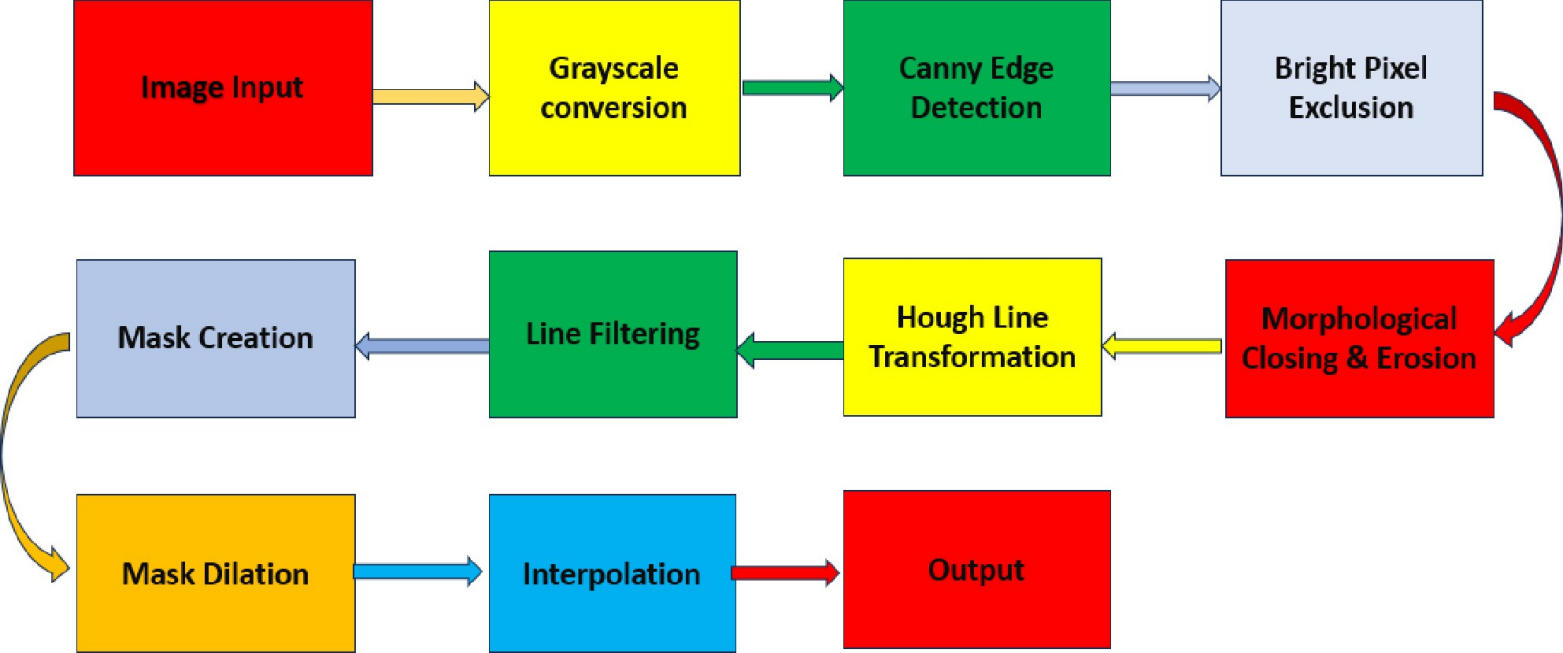
Hair removal architecture.

First, the image is converted to grayscale, removing any color information that might interfere with edge detection. Then, the Canny edge detector is applied to identify sharp transitions in brightness, which likely represent edges in the hair. However, very bright pixels are excluded as they’re unlikely to be hair. To refine the edges further, a morphological closing operation is performed, which helps to connect small gaps and smooth the edges. Following this, erosion is applied to remove isolated, noisy pixels that might be falsely identified as edges. Next, the probabilistic Hough Line Transform is used to identify lines within the refined edges. These lines likely correspond to the strands of hair. However, very short or long lines are discarded as they’re probably not hair. Finally, a mask is created based on the coordinates of the remaining lines. This mask is then dilated to slightly expand the hair region. Lastly, the pixels within the dilated mask are interpolated using the median value of their neighboring pixels, effectively filling in any gaps within the hair and creating a smoother result. Proposed architecture shown in Fig 2.

### ResUNet++ based skin lesion segmentation

**Fig 3:**
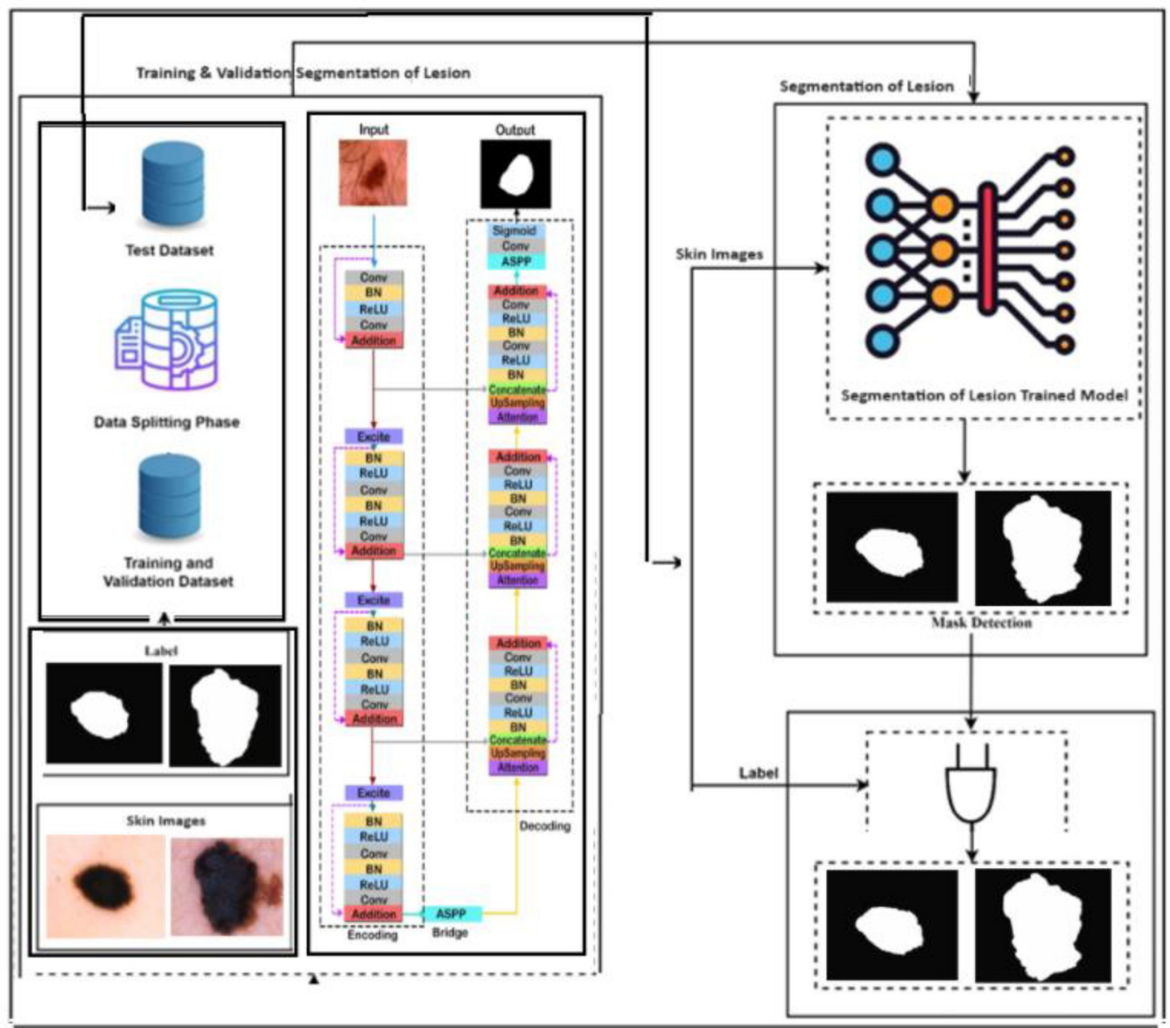
Proposed ResUNet++ and modified AlexNet-RF based segmentation and classification of skin lesion.

Lesion segmentation is the second stage of the skin lesion classification using ResUNet++ based deep learning model for segmentation is shown in Fig 3. In this phase, ResUNet++ based lesion segmentation architecture is applied to the segment lesion from skin Dermoscopic images. The segmentation phase consists of two steps: training & validation of segmentation of lesion step and segmentation of lesion step. In the segmentation of lesion step, ResUNet++ based deep learning model trained and validated on HAM dataset. In the second step, the ResUNet++ based deep learning model for lesion segmentation predicts the mask from the test Dermoscopic images dataset and by using the predicted mask, the lesion is extracted. In this phase, the dataset consists of Dermoscopic images with labels that are used as input to the proposed method for segmentation. The Dermoscopic images dataset is divided into training and testing.

The masks of the assessment images are predicted by the segmentation of the lesion model. By employing the predicted mask of segments, the lesion is ultimately segmented from images. Ranneberger et al. [25] developed the ResUNet++ architecture for medical image segmentation in 2015. As illustrated in Fig 3, the encoder, decoder, and bypass connection constituted the three fundamental components of the ResUNet++ architecture. Built specifically for semantic segmentation tasks such as skin lesion segmentation, ResUNet++ is a dense convolutional block-based deep learning architecture that utilizes residual learning. An explanation of the mathematical model is provided in the subsequent manner:

#### Essential components

The core component of ResUNet++ is the residual unit, which implements the concept of residual learning. Denoted as F(x), the transformation performed by the residual block on the input x represents the residual unit. As the result of, the unit’s output is produced by following Eq (1).


y=F(x)+x (1)

Instead of attempting a direct translation from input to output, this architecture facilitates the network’s acquisition of residual functions. It assists in mitigating the challenge that deep neural networks might face when gradients vanish.

#### Dense Convolutional Block (DCB)

The ResUNet++ enhances the feature extraction process with the use of Dense Convolutional Block that were introduced. Convolution layers (Conv) are building blocks of the DCB, but are different among themselves by growth rate(k).

Included in a DCB:

Let x0 be the input to the DCB.

For each layer i (1 to L), the output (xi) is calculated as follows by Eq (2)

x‐i=H(concat(x‐(i−1),Conv(x‐(i−1),k))) (2)

A rectified linear unit (ReLU) activation and batch normalization (BN) layer are often combined into a composite function denoted by H.

The process of concatenation contains combining the feature maps from the previous layer (x‐(i−1)) with the output of the current convolutional layer (Conv(x‐(i−1),k)).

The end result of the modular architecture of DCB is the high degree of interconnectivity, entailing the information exchange and the feature reuse between the layers, which may lead to the formation of the improved feature representations.

The overall ResUNet++ architecture can be visualized as a stack of convolutional blocks with skip connections. Here’s a simplified breakdown:

1. *Initial Convolution*:
  An initial convolutional layer is employed to extract the low-level features of the input image.
2. *Bottleneck Blocks*:
  In order to reduce computational expenses while maintaining representational capacity, bottleneck blocks, such as 1x1 convolutions, can be implemented as a series of residual units.
3. *Dense Convolutional Blocks (DCBs)*:
  An ascending growth rate is applied to a sequence of DCBs in order to progressively extract more complex features.
4. *Transition Blocks*:
  Pooling algorithms, such as max pooling, may be implemented within these blocks to augment the channel count of feature maps while reducing their spatial resolution. This is typically the location where encoder bypass connections are appended.
5. *Decoder Path (Up-sampling)*:
  The decoder utilizes a variety of techniques, including transposed convolutions (deconvolutions) and bilinear interpolation, to up sample feature maps and increase their spatial resolution.
6. *Output Layer*:
  A final convolutional layer is employed to predict the class probabilities for each pixel. This layer possesses a 1x1 kernel size and an equal number of filters as the number of segmentation classes. To convert these probabilities into a class mask, one may utilize the SoftMax function.

Firstly, Dermoscopic images are obtain from the freely accessible skin tumor namely HAM dataset. These images are presuming that as input to the data preprocessing stages to tune the data for the modeling stages. In data preprocessing stages, the input images are modified while noise and outliers are cleared. Next phase segmentation. For segmentation we use ResUNet++ architecture.

Our proposed ResUNet++ architecture [13] consists on several key point that integrated to increase performance of model. Main key component of this model includes Squeeze and Excitation blocks, an attention and Residual Block and one is Atrous Spatial Pyramidal Pooling (ASPP). Residual block plays a vital role in modifying the degradation problem in each encoder block while at the same time it decreases the computational directly above capably transmission dense features maps to consequent. ResUNet++ architecture has each stem consist on three encoder, three decoder and one ASPP block. Fig 3 shows block diagram of structure of ResUNet++ architecture.

Each convolution block of ResUNet architecture consist on single convolutional layer, a batch has normalization layer and a activation function name as a rectified linear unit (ReLU). In first layer of convolutional, encoder block and spatial dimension of resulting features maps are slitted through the employment of stride convolutional layers. Output of preceding encoder unit is forwarded to squeeze and excitation block as input. Atrous Spatial Pyramidal Pooling serves to extend the field of view for each filter, linking mechanism is functioning. Up sampling low level features maps services to neighbors sampling, then it fused with the conforming features maps from the encoder path. For final segmentation map generate, decoder block output undergoes through the Atrous Spatial Pyramidal Pooling that followed by single convolutional layer with sigmoid activation function.

### Modified AlexNet-Random Forest based classification of skin lesion

**Fig 4:**
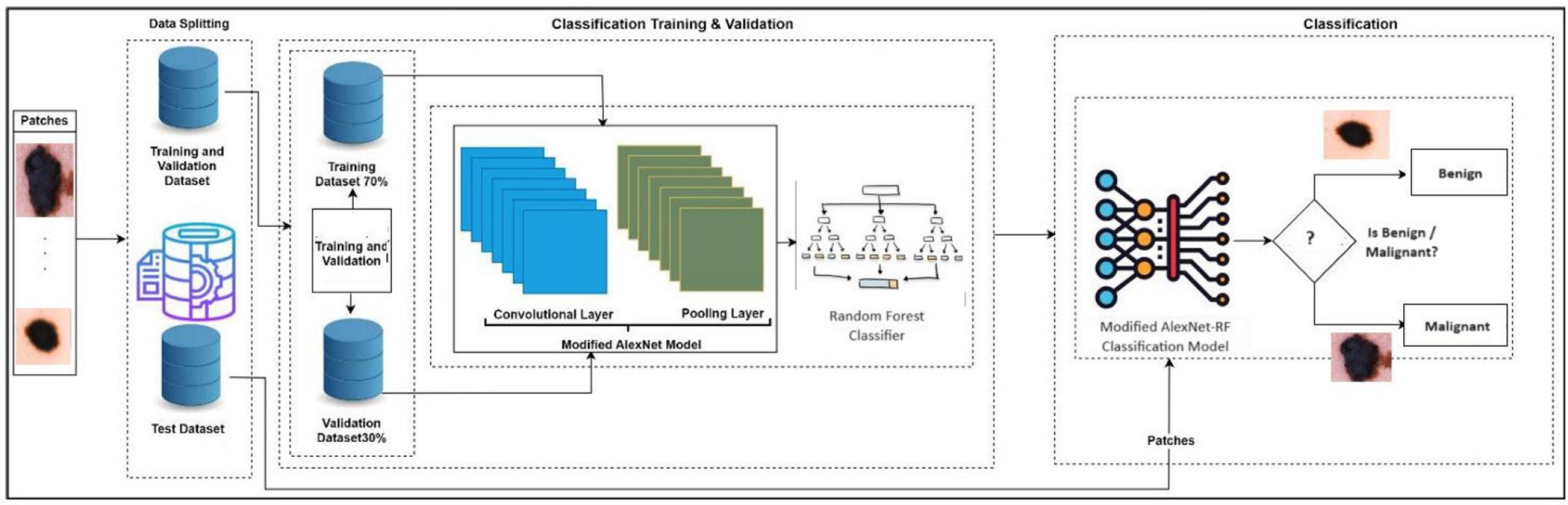
Proposed modified AlexNet-RF based classification of skin lesion.

The AlexNet-RF model is structured into two main phases: training and validation, illustrated in Fig 4. Initially, Dermoscopic images are sourced from the publicly available HAM dataset. These images undergo preprocessing to prepare them for the modeling phase. During preprocessing, the input images are reshaped, and noise and outliers are eliminated. Additionally, the input patch size is segmented into three dimensions: 16 × 16, 32 × 32, and 48 × 48. Subsequently, the preprocessed data is divided into training and validation sets, with 80% assigned for training and 20% for validation. This segmented data is then passed on to the data splitting phase.

There are two layers in training data: application and performance layers. In the application layer, we have extracted features using the suggested AlexNet-RF modified convolutional neural network. Crucial data is extracted from the input photos during the features extraction procedure and sent to the following stage. Three optimizers Using hyperparameters such as 0.0001 learning rate, 50 batch size, and 200 epochs, adaptive moment estimation (Adam), the root mean square CMC, 2023, vol.74, no.1 2045 propagation (RMSprop), and stochastic gradient descent (SGD) are employed.

In the performance layer, the suggested AlexNet-RF model was assessed for accuracy and miss classification rate. The core component of a CNN is the convolution layer, which is in charge of extracting significant characteristics from the input data. Convolutional processes, symbolized with this symbol (*) are carried out in convolutional layers, and the results of these operations cannot be applied to an image without a filter. Feature maps or stimulation maps are the results of the convolutional algorithm. Eq (3) displays the convolutional operation [32].


(U*V)(x,y)=pZ(x,y) (3)

q U (y + q, x + p)V(p, q) (1), where Z is the output feature map, V is the filter size for p × q, and U is the input matrix (image). The map of features Z is created after the input U and filter V are convolved. The mathematical representation of this convolutional operation is U × V. The convolution layer’s output is sent to a nonlinear activation function (AF). The function of activation gives a network more non-linear behavior To eliminate linearity and standardize the networks data, a number of nonlinear activation functions, like soft max, sigmoid, hyperbolic tangent (Tanh), and rectified linear unit (ReLU), can be applied to the activation map. In this work, activation is calculated using the ReLU activation function. The ReLU output is 0 if the input is zero or less below zero. ReLU [37] is a mathematical illustration of what Eq (4) gives.


max(0,z)=f(z) (4)

The layers of the Pooling & Convolution layer come after each other in the convolutional neural network. utilized to reduce the feature map’s dimensions while preserving crucial data; this technique is referred to as down sampling. The pooling layer uses a variety of procedures, including min pooling, max pooling, average pooling, and sum pooling, to minimize the size of the activating map. Important information is preserved in the pooling layer, but the activation map’s size is shrunk.

**Fig 5:**
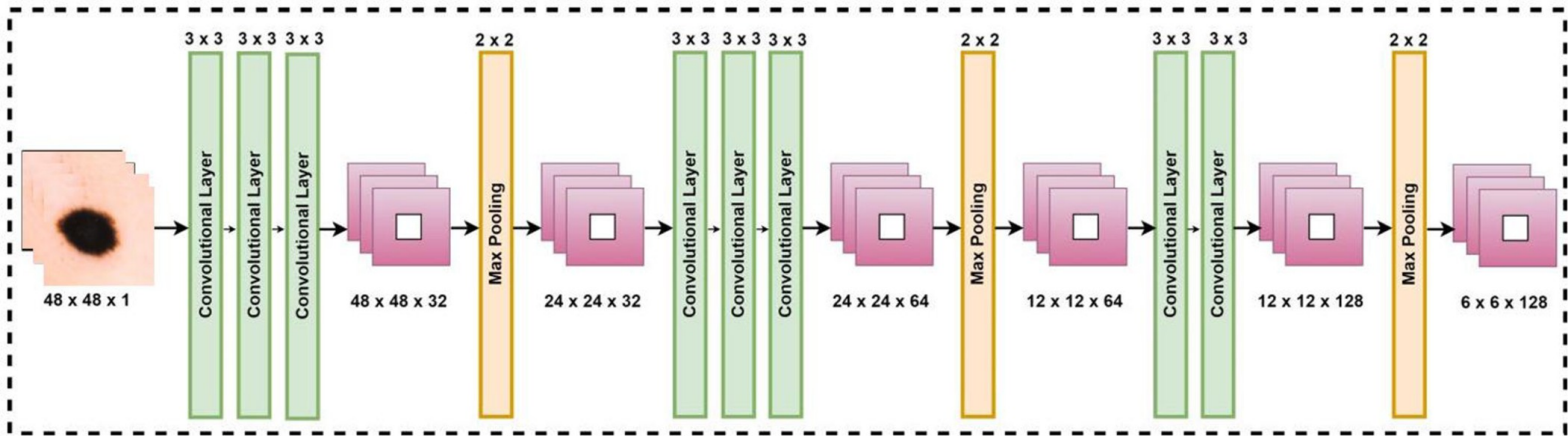
Modified AlexNet based classification of skin lesion.

The flattened feature map is passed to the completely connected layer first. The feature map matrix becomes a lengthy vector after flattening. In the convolutional layer, 80% of the preprocessed dermoscopic image data is used for convolutional operations. A total of twelve layers are used in the proposed AlexNet-RF, consisting of three pooling, two fully connected, and seven layers that are convolutional. Fig 2 shows the suggested AlexNet-RF CNN network topology for tumor identification. The suggested network architecture accepts grayscale picture input sizes of 16 × 16, 32 × 32, and 48 × 48. Fig 5 illustrates this with an image size of 32 × 32 × 1. With the same padding and a kernel size of 3 x 3, a total of 32 filters are applied in the first pair of convolutional layers.

Using the ReLU activation function, the recommended model’s nonlinearity is eliminated. A max-pooling layer comes after layers of convolutional neural networks. The max-pooling layer adopts a filter size of 2 × 2 with a stride of 2, resulting in a reduction in image size to 16 × 16 × 32.

Then, 64 filters were applied by a pair of convolutional layers with the same padding and kernel size of 3 × 3. Function of ReLU activation. After applying the 2 × 2 kernel capacity and speed for the second maximum pooling layer, the image size increases to 8 × 8 × 64. The final trio of convolutional layers utilize 128 filters, a 3 x 3 kernel, and the activation function of ReLU. After applying two strides and a kernel size of 2 × 2, the 3rd maximum pooling layer’s size increases to 4 × 4 × 128. The third max-pooling layer is then applied, flattening the multidimensional supply to a 1D vector with a size of 2048 × 1. 2046 C.

A Fully Connected (FC) layer is used for carrying out the classification operations of a convolutional neural network (CNN) following features extraction [38]. In an FC layer of a standard neural network, every neuron in the layer above is coupled to every other neuron in the layer below. The fully linked layer’s result is sent to AF, which generates class scores. The two methods most frequently used to calculate classification purposes are SoftMax while helping vector machine models. To attain the best efficiency in classifying lung cancer as benign or malignant, the randon forest (RF) classifier is utilized in the AlexNet-RF model. As a result, the functionality layer receives these results.

To train the algorithm for deep learning requires an enormous amount of processing power and time. For this reason, various optimization tools are important.

Statistical measures like accuracy and the miss categorization rate of the AlexNet-RF are included in the performance layer. The suggested AlexNet-RF model must be trained if the learning criteria does not match the needs. If the learning criteria does match the requirements, the model and results are kept on the cloud for use in the future. The proposed AlexNet-RF training phase ends when it is ready for validation.

20% of the validation data is sent to the trained AlexNet-RF version during the validation step, which involves downloading the trained model from the cloud and evaluating the suggested model. The trained model indicates malignant skin cancer if it finds a cancer nodule; if lung cancer fails to detect any cancer nodules, the model predicts benign skin cancer.

A Fully Connected (FC) layer is used for carrying out the classification operations of a convolutional neural network (CNN) following features extraction [38]. In an FC layer of a standard neural network, every neuron in the layer above is coupled to every other neuron in the layer below. The fully linked layer’s result is sent to AF, which generates class scores. The two methods most frequently used to calculate classification purposes are SoftMax while helping vector machine models. To attain the best efficiency in classifying lung cancer as benign or malignant, the random forest (RF) classifier is utilized in the AlexNet-RF model. As a result, the functionality layer receives these results.

To train the algorithm for deep learning requires an enormous amount of processing power and time. For this reason, various optimization tools are important.

Statistical measures like accuracy and the miss categorization rate of the AlexNet-RF are included in the performance layer. The suggested AlexNet-RF model must be trained if the learning criteria does not match the needs. If the learning criteria does match the requirements, the model and results are kept on the cloud for use in the future. The proposed AlexNet-RF training phase ends when it is ready for validation. 20% of the validation data is sent to the trained AlexNet-RF version during the validation step, which involves downloading the trained model from the cloud and evaluating the suggested model. The trained model indicates malignant skin cancer if it finds a cancer nodule; if lung cancer fails to detect any cancer nodules, the model predicts benign skin cancer.

## Results and discussions

### Dataset

**Fig 6:**
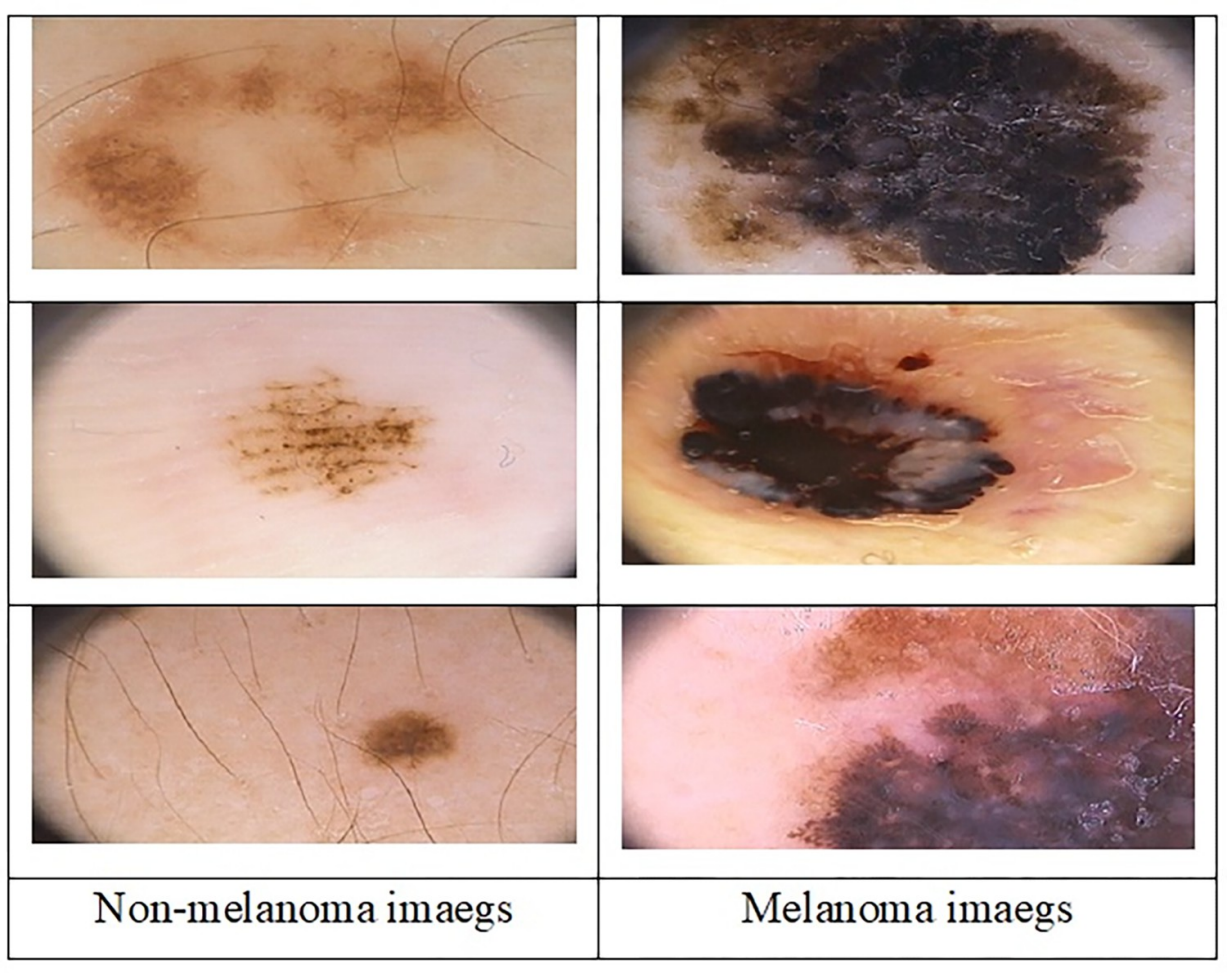
Sample of different Dermoscopic images from HAM10000 database.

To evaluate the proposed technique, we used a different three datasets PH2, ISIC2019 and HAM1000 that publicly available on googles. Some images shown in Fig 6.

### Performance measures

Utilizing the Dice Similarity Coefficient and Region Base Coincidence Criteria, the efficacy of the segmentation process is evaluated and confirmed.

Jaccard Similarity Index for Region Coincidence

$$P(R,A)=\frac{\left|A\cap R\right|}{\left|A\cup R\right|},\;0\leq P(R,A)\leq 1$$ (5)

In Eq 5 where R is the discovered segment foreground structure and A is the ground truth. The degree to which the ground truth structure is discovered is indicated by the factor |A∩R|. The accuracy measure and normalization are displayed by |A∪R|. The optimal matching or similarity between the system’s estimated area and the ground truth is indicated by the value 1.

Dice Similarity Coefficient

$${DSC}_{\left(A,R\right)}=\frac{2|A\cap R|}{\left|A|+|R\right|}$$ (6)

In Eq 6 where R represents the area that the expert has identified as the ground truth and A represents the segmentation pixels that the proposed system has recovered. A DSC value falls within the range of zero to one. The segmentation method exhibits superior performance when the DSC values approach 1, the optimal value.

**Fig 7:**
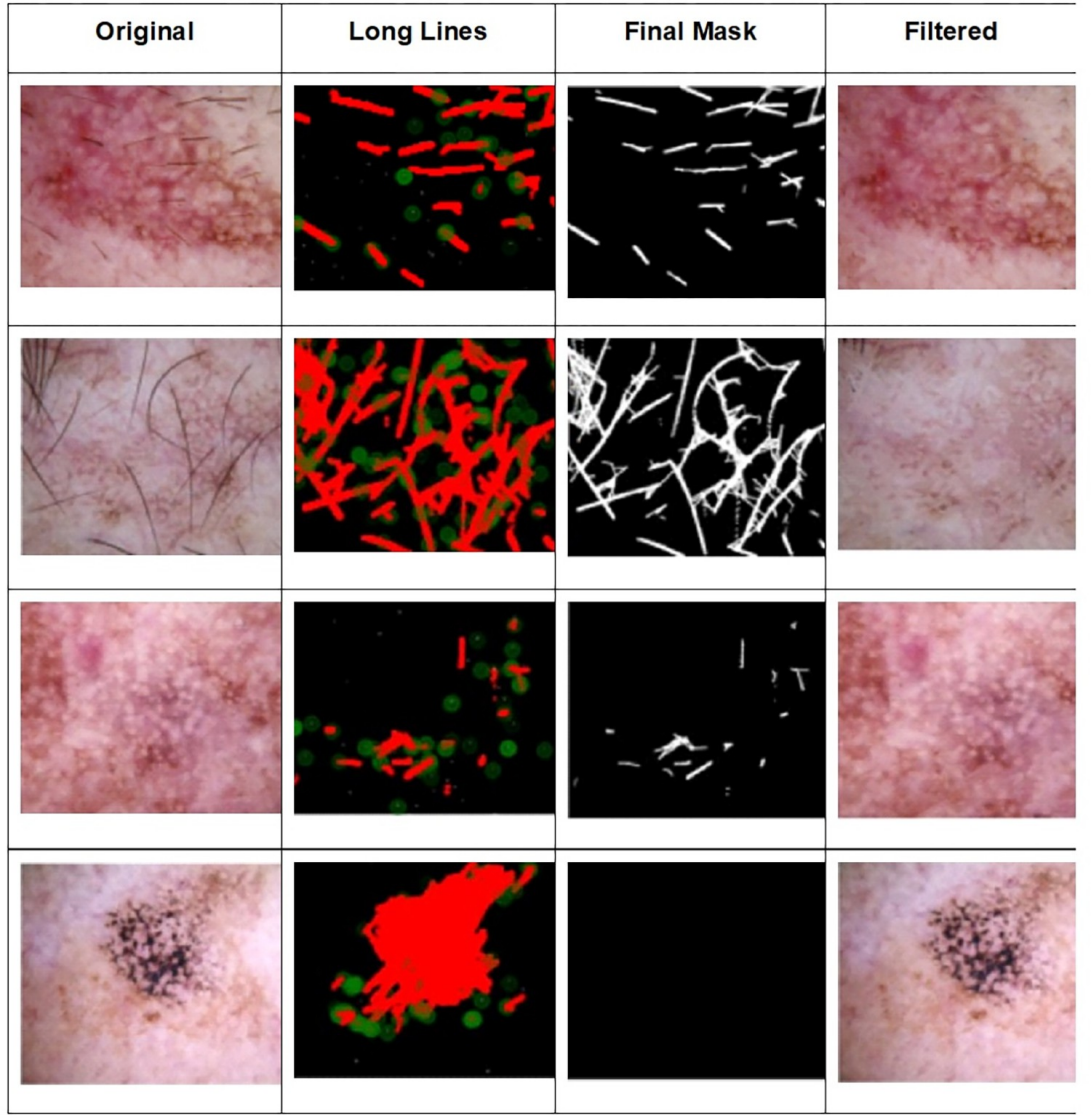
Morphology-Hough transform based hair removal from skin images.

To assess the performance of these distinct feature sets three key metrics. Accuracy, sensitivity and specificity have been measured. The results of Morphology-Hough Transform based Hair Removal from Skin Images are shown in Fig 7.

To check the performance of the classifiers is following performance measured:

**Accuracy.** The ratio of correctly classified instances (true positive + true negatives) to the total number of instances. Mathematically, it is represented as

Accuracy=(TN+TP)/(TN+FP+TP+FN) (7)

#### Sensitivity

Also known as recall, it measures the ability of the classifier to correctly identify instances of a specific class. For instance, in the context of malignant masses. The sensitivity is calculated as

Sensitivity=TP/(TP+FN) (8)

#### Specificity

It gauges the classifier’s capacity to correctly identify instances of the other class (benign masses. In this case). Specifically, specificity Is calculated as

Specificity=TN/(TN+FP) (9)

These performances metrics, including accuracy, sensitivity, and specificity, provide valuable insights into the classifier’s effectiveness in distinguishing between classes.

**Fig 8:**
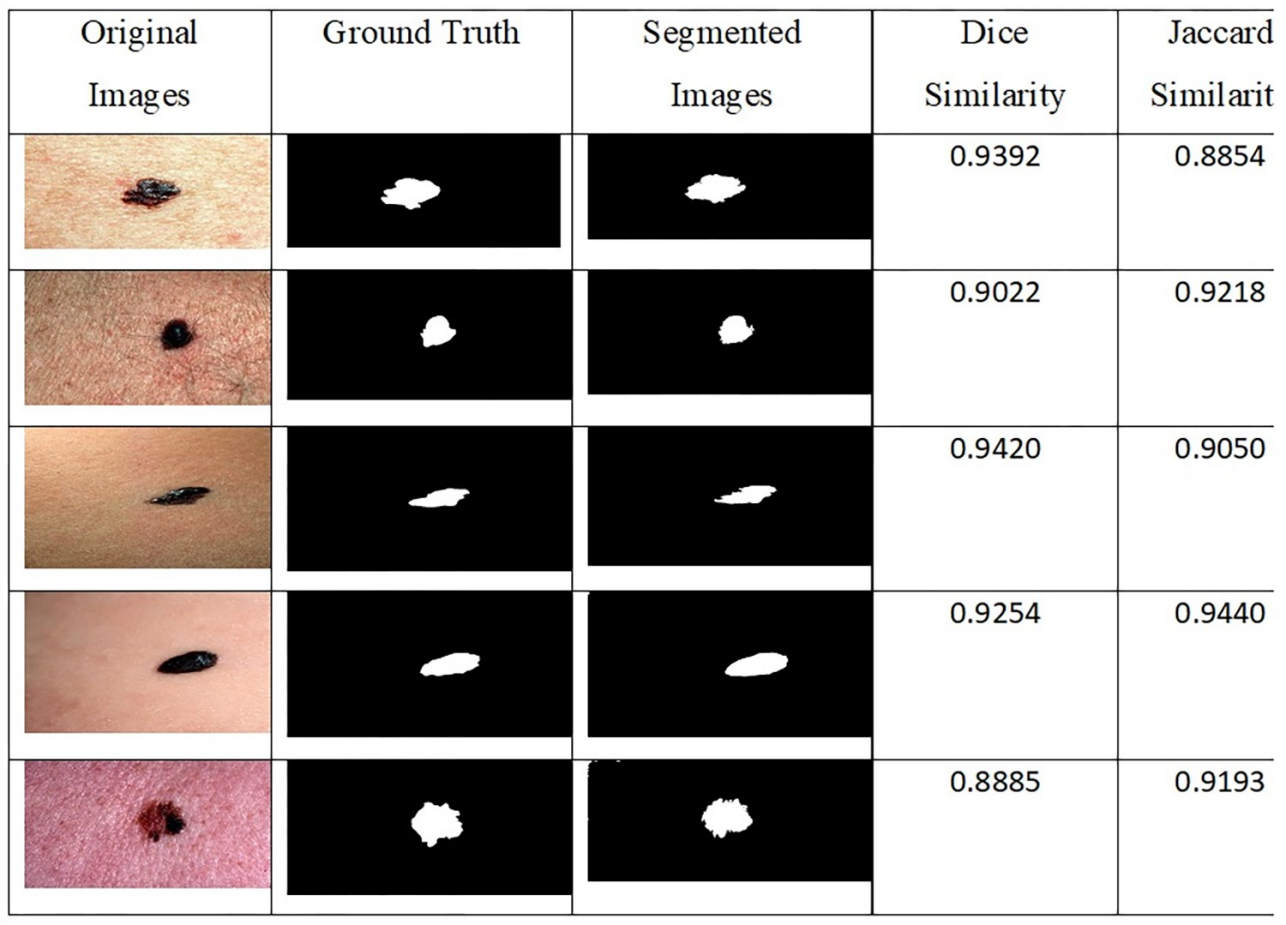
Segmentation results applied to the input images.

The results of images segmentation are shown in Fig 8.

**Fig 9:**
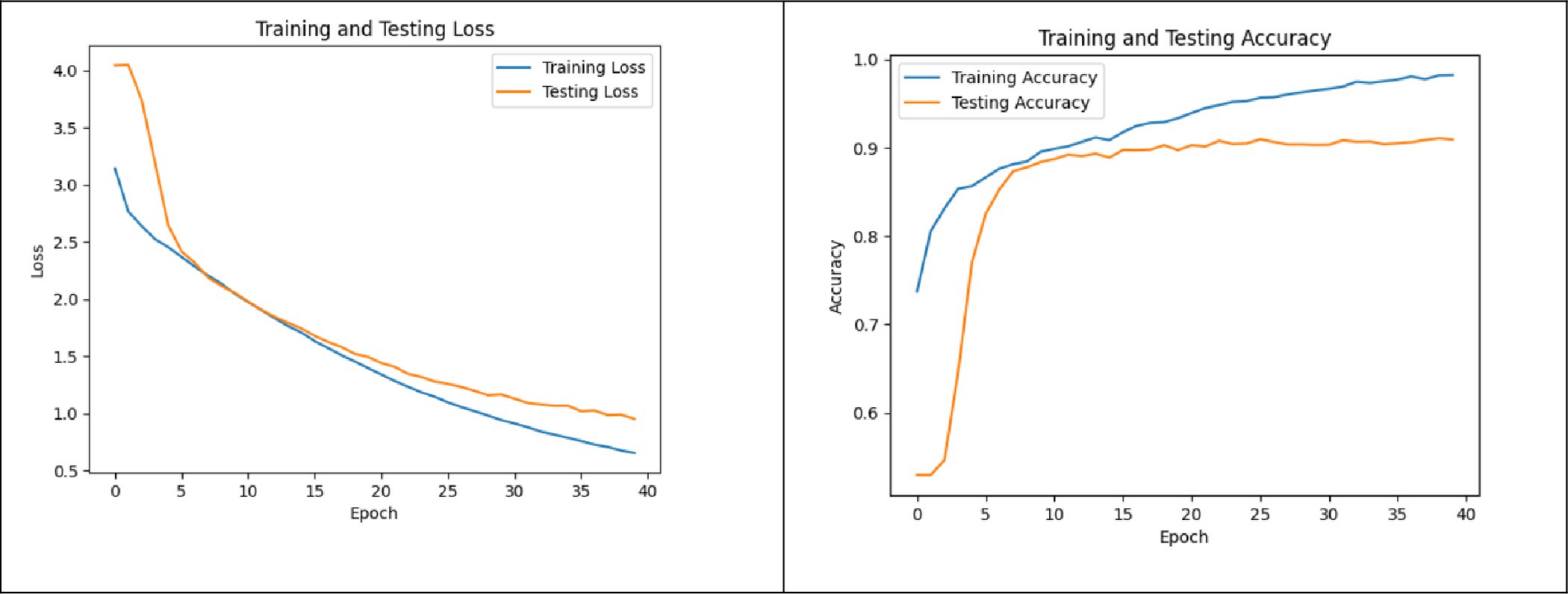
The scatter plots showed distinct behaviors of different features.

Upon analyzing the result, it is observed that ST consistently achieves slightly higher accuracy, sensitivity, and specificity metrics compared to the individual feature set. The scatter plots depicted in the Fig 9 below illustrate distinct behaviors of different features.

Specifically, the first and second rows exhibit overlapping features, making classification challenging, in contrast to the feature shown in the third row, where features are clearly separated with minimal overlap.

**Fig 10:**
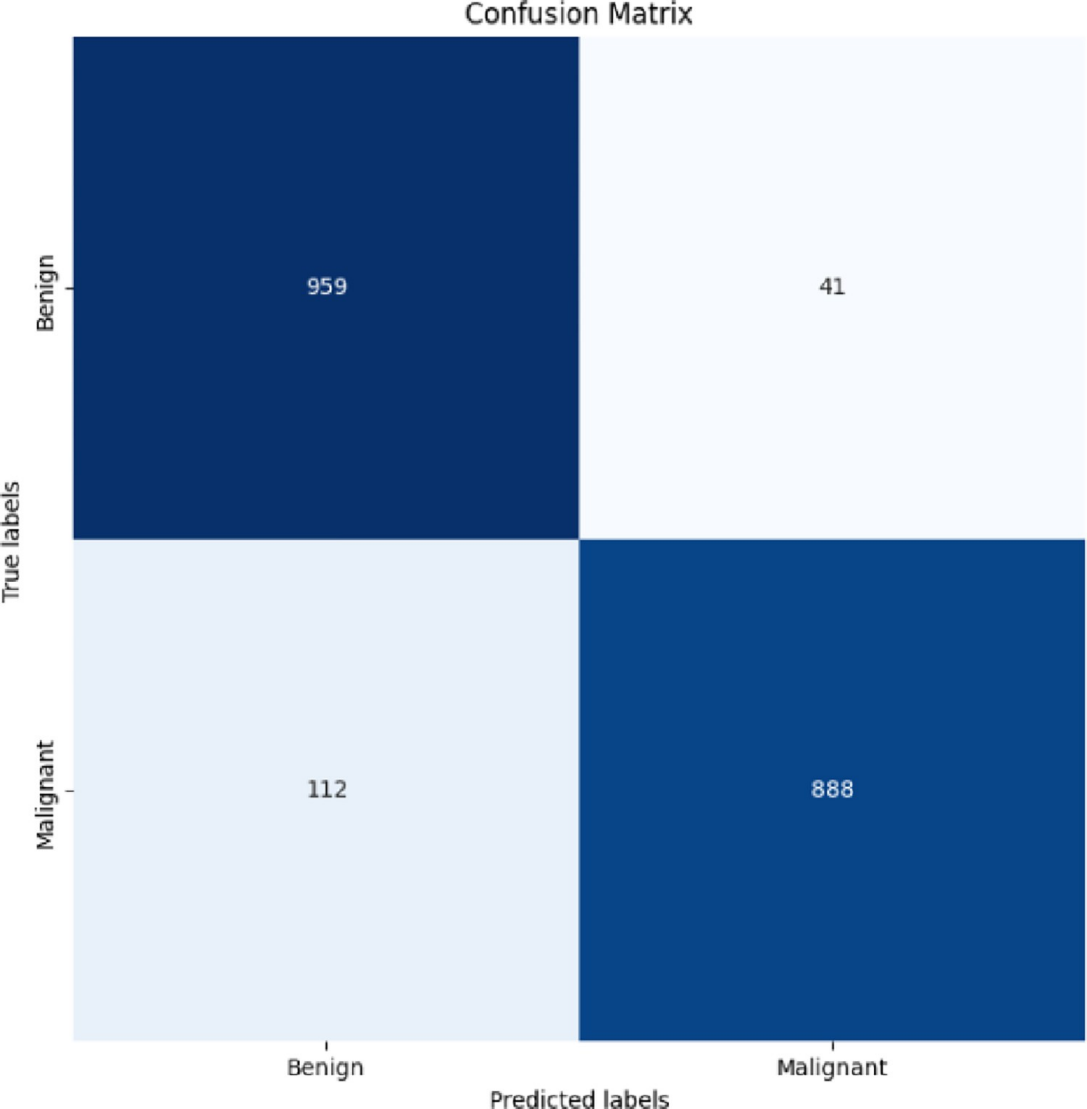
Confusion matrix of our proposed modified AlexNet-RF classification.

Fig 10 showed the Confusion matrix of our proposed modified AlexNet-RF classification that is outstanding performance.

Table 2 showed our experimental outcome for classification compares with the existing technique of deep learning model. Our results showed a effective and efficient over existing techniques. Our proposed ResUNet++—AlexNet-RF showed on HAM10000 dataset 92%acciracy ISIC2019 93.4% and PH2 94.2%

**Table 2:** Skin lesion classification performance using deep learning methods.

Reference	Dataset	Method	Accuracy
Tan et al. [26]	ISIC 2018	CNN	83.2%
Bassel et al. [27]	ISIC	Resnet50, Xception, and VGG16	90.9%
Fraiwan and Faouri [28]	HAM10000	thirteen CNN architectures with DensNet201	82.9%
Alam et al. [29]	HAM10000	S2C-DeLeNet	91.03%
Jain et al. [30]	HAM10000	Transfer learning-based VGG19, InceptionV3, ResNet50, Xception, and MobileNet	90.48%
Bechelli and Delhommelle [31]	HAM10000 AND KAGLE DATA SETS	CNN, pre-trained VGG-16, Xception, ResNet50	88%
Abdar et al. [32]	ISIC 2019	ResNet152V2, MobileNetV2, SDenseNet20	89%
Garg et al. [33]	ISIC	ABCD Rule Technique	91%
Taufiq et al. [34]	Skin Photos	m-skin App with SVM	80%
Zhuang et al. [35]	ISIC2019	CS-AF with 12 CNNs	77.47%
Proposed	Ham10000	ResUNet++—AlexNet-RF	92%
	ISIC2019	ResUNet++—AlexNet-RF	93.4%
	Ph2	ResUNet++—AlexNet-RF	94.2%

Table 3 showed experimental results of skin cancer segmentation using modified ResUNet++—AlexNet-RF for segmentation. Our proposed model have shown HAM10000 dataset 92%acciracy ISIC2019 93.4% and PH2 94.2% that is outstanding performance.

**Table 3:** Skin lesion segmentation performance using deep learning methods.

Reference	Dataset	Method	Accuracy
D. Popescu et al. [36]	HAM10000	CNN Models	86.71%
N. C. F. Codella et al., [37]	900 training and 379 testing images	NA.	76%
A. Wibowo et al. [38]	ISIC-2017, PH2	MobileNetV3-UNet	88.00%
L. Hoang, S. et al. [39]	ISIC 2019, HAM10000	Wide-shuffleNet	86.33%
M. Aminur, et al. [40]	HAM10000	Different CNN models InceptionV3	89.81%
M. A. Al-masni, et al. [41]	ISIC-2017	DenseNet-201	81.29%
B. Ahmad et al. [42]	NA.	ResNet-152,InceptionResNet-V2	87.42%
Proposed	HAM1000	ResUNet++—AlexNet-RF	92%
	ISIC2019	ResUNet++—AlexNet-RF	94.2%
	PH2	ResUNet++—AlexNet-RF	93.8%

## Conclusion

This paper proposes a novel methodology for skin lesion segmentation and classification using a hybrid deep learning approach. The proposed method consists of three key phases: preprocessing, lesion segmentation, and lesion classification. Hair can obstruct lesion boundaries. A morphology-based hair removal technique likely improves segmentation accuracy by creating a cleaner image for analysis compared to methods that don’t address hair. So during preprocessing, a morphology-based technique is employed for efficient hair removal.

Segmentation isolates the lesion from surrounding healthy skin. This allows the classification stage to focus solely on the relevant region, eliminating background noise and irrelevant features that could impact classification accuracy. By separating the lesion, the model can analyze its characteristics (color, texture, borders) more precisely for accurate classification as benign or malignant. Accurate segmentation ensures these features are extracted only from the lesion itself, leading to a more reliable feature set for the classification model. For accurate lesion segmentation, a state-of-the-art deep learning architecture, ResUNet++, is utilized. Following segmentation, a modified AlexNet-Random Forest (AlexNet-RF) classifier is implemented for robust lesion classification. The proposed hybrid deep learning model is extensively evaluated on the Ham10000 dataset, a benchmark dataset for skin lesion analysis. The achieved results demonstrate that the proposed method outperforms existing methods, achieving superior performance in both segmentation and classification tasks. By utilizing ResUNet++, a well-established deep learning architecture known for its accuracy in medical image segmentation, your method likely achieves superior lesion segmentation compared to simpler approaches. This translates to a more precise analysis in the classification stage. The combination of AlexNet, a powerful deep learning model for feature extraction, with a Random Forest classifier leverages the strengths of both techniques. AlexNet can capture intricate features, while the Random Forest improves robustness and reduces overfitting, potentially leading to more accurate classification compared to using a single model.
